# Antibacterial effect of various herbal root canal irrigants

**DOI:** 10.6026/973206300210165

**Published:** 2025-02-28

**Authors:** Vathsala N., Saikiran Bahadur, Aseem Sharma, Nisha Gupta, Ahmed Ali Ahmed Almuntashiri, Reshma Rajan, Arpita Mohapatra, Awadhesh Gupta

**Affiliations:** 1 Department of Conservative Dentistry and Endodontics, The Oxford Dental College and Hospital, Bangalore, Karnataka, India; 2 Departments of Prosthodontics, Springfield Dental, Springfield, Massachussets, USA; 3 Department of Orthodontics and Dentofacial Orthopedics, Himachal Institute of Dental Sciences (HIDS), Paonta Sahib, Himachal Pradesh, India; 4 Departments of Conservative Dentistry and Endodontics, ESIC Dental College and Hospital, Delhi, India; 5 Department of Public Health, College of Applied Medical Sciences, Qassim University, Buraydah-51452, PO Box-6666, Saudi Arabia; 6 Department of Pedodontics and Preventive Dentistry, Malabar Dental College and Research Centre, Kerala, India; 7 Department of Periodontology, Kalinga Institute of Dental Sciences, Bhubaneswar, Odisha, India; 8 Department of Oral Pathology and Microbiology, DJ College of Dental Sciences and Research, Modinagar, Ghaziabad, Uttar Pradesh, India

**Keywords:** Antibacterial, herbal, irrigants, root canal

## Abstract

Root canal irrigation is crucial in endodontics. Therefore, it is of interest to evaluate the antibacterial efficacy of various herbal root canal irrigants (Triphala, Neem (*Azadiracta indica*, green tea and *Curcuma longa* (Turmeric)) against *Enterococcus faecalis*. Antimicrobial efficacy of the herbal irrigants and sodium hypochlorite was done using a brain-heart infusion method. The tested herbal irrigants had antibacterial efficacy against *E faecalis*. Hence, these herbal irrigants are alternative to sodium hypochlorite.

## Background:

Endodontic therapy aims to control the disease in the periapical area by eliminating all necrotic or living tissue, microbes and microbial by products from the root canal system [1, 2]. A chemical solution and mechanical instrumentation are utilised in the root canal space in two processes that are usually referred to as "chemo mechanical" preparation in order to clean the root canal. The oral cavity contains a large number of bacteria, but because oxygen and nutrients are scarce in endodontic infections, only a small number of bacterial species are present. Maximum disinfection is necessary for the root canal procedure to be successful in the long run [3]. Accumulated debris and microorganisms cannot be removed alone with instrumentation [2]. Therefore, in order to completely remove bacterial and necrotic debris from intricate root canal networks, mechanical instrumentation should be used in conjunction with sufficient irrigants solutions [4, 5]. Numerous root irrigants were recommended, including Bio-Pure MTAD, QMixTM, 2% chlorhexidine, sodium hypochlorite (NaOCl) and regular saline [4]. Many chemical irrigants used in endodontic have antimicrobial activity [1]. Chemical irrigants have disadvantages such cytotoxicity, medication resistance and microbial resistance, despite their effectiveness in root canal irrigation [4]. To overcome these side effects herbal alternatives such as; neem, turmeric, tulsi, triphala, aleovera, green tea have been tried [1, 6]. Herbal extracts are more economic than chemical ones and have antibacterial, antifungal, analgesic and anti-inflammatory properties [1, 7 and 8]. NaOCl is the irrigation solution of choice in routine endodontic practice. NaOCl has wide range of antimicrobial activity, excellent tissue dissolution capability, accessibility & relatively lower cost. Its drawbacks are; strong bleach odor, allergic reactions to the ocular & nasal mucosa. When NaOCl is extruded beyond the apex, it causes severe inflammatory responses, which ultimately destroys apical essential tissues [8]. Neem or Azadirachta indica has anti-inflammatory, antiviral, antifungal and antibacterial qualities [9]. Neem extract has anti plaque efficacy and it can be used as root canal irrigants and to reduce periodontal pathogens [7]. The active ingredients in neem, such as nimbinin, azadirachtin and nimbidin, give it its special therapeutic qualities [9].

The word Triphala Comes from the Sanskrit words tri-three, phala - fruits, a polyhedral medicine consisting of an equiproportional mixture of powder of 3 medicinal fruits, namely *Emblicaofficinalis*, *Terminalia chebula*, *Terminalia belerica*. Tannic acid content of it has antibacterial action. Triphala holds promise to remove the smear layer without affecting the microhardness of root dentin. Green tea has antifungal activity, smear layer removing capability [8]. Green tea polyphenols have significant anti-cariogenic, antioxidant, thermogenic, anti-inflammatory, probiotic andante-microbial properties. *Curcuma longa* (Turmeric) is an Indian spice possesses anti-inflammatory, antioxidant, antimicrobial, anticancer activity, anti-malarial and hepatocellular properties. It is effective against gram positive and gram negative bacteria [10]. Primary endodontic infection comprises a mixed community of bacterial species. Microbiota isolated from clinically asymptomatic teeth is completely different from microbiota isolated from clinically symptomatic teeth. Aerobic, facultative organisms and anaerobic microbes were identified in infected deciduous root canals. *Enterococcus faecalis*, *Porphyromonas gingivalis* and *Treponema denticola* are reportedly the most prevalent species isolated from deciduous root canals. Facultative organisms are the main culprit for the pathogenesis of the disease process [11]. *Enterococcus faecalis* is the most prevalent pathogenic microorganism in root canal therapy. It is possible for *E. Faecalis* to thrive in extremely alkaline environments [12]. 80-90% of enter coccal infections are caused by *Enterococcus faecalis*, an anaerobic gram-positive bacterium that is typically isolated from unsuccessful root canals. Its capacity to infiltrate dentinal tubules and its virulence, which is ascribed to its resistance to intracanal medications, make it a crucial factor in the on-going failure of endodontic therapy [9]. Eliminating these bacteria is extremely challenging due to its resistance to the antimicrobial actions of calcium hydroxide (CH), penetration into the dentinal tubules and adherence to dentin [13]. *E. Faecalis* is most common and more resistant to endodontic treatment because it is capable of entering the dentinal tubules and adheres to collagen in the presence of serum, causing root canal failures [3]. Eradication of microorganisms from the root canal is must for successful endodontic procedure. Therefore, it is of interest to evaluate different herbal root canal irrigants against *Enterococcus faecalis*.

## Materials and Methods:

This *in vitro* study was conducted in the Departments of Conservative Dentistry & Endodontics and Department of Microbiology. Extracted single rooted teeth due to orthodontics purpose without any pathology were included for the study. The selected teeth were disinfected with NaOCl then decoronated at the cement enamel junction using the diamond disc with copious water. Root canal was done for all the teeth followed by saline irrigation then apical foramen of all the specimens was sealed with auto polymerizing acrylic resin to prevent bacterial leakage. Then all the teeth specimens were autoclaved. Sterilised root canals were re-infected using *E. Faecalis* ATCC 29212 microbial strains. Coronal access and root apices of each root section was sealed and incubated at 37°C for 48 h. Following the contamination procedure, specimens were assigned into five groups with 10 samples in each according to the irrigant used as; Group I- Sodium hypochlorite, Group II- triphala, Group III- neem, Group IV- turmeric and Group V-green tea.

## Preparation of herbal extracts irrigants:

After being harvested from the neem trees, fresh leaves were cleaned in sterile distilled water. The process was repeated after the prepared neem extract was run through muslin cloth to remove any coarse residue. After obtaining two extracts, they were combined and filtered using quick filter paper. To create an irrigation solution with a 5 mg/ml concentration, triphala powder (IMPCOPS Ltd., Chennai, India) was dissolved in 10% dimethyl-sulfoxide (SD Fine Chemicals, Chennai, India). Turmeric irrigants was prepared by dissolving fine powder of turmeric in 10% dimethyl-sulfoxide. Green tea irrigating solution prepared form fine powder of green tea leaves. Root canals were irrigated with each irrigants and bacterial swab was collected using a sterile paper points before irrigation and after irrigation with paper point and tested for antibacterial efficacy using brain-heart infusion (BHI) broth (Difco Laboratories, Detroit, MI, USA). Colony forming unit of *E. Faecalis* was calculated and obtained data was statistically analysed using one way ANOVA test.

## Results and Discussion:

Table 1 indicates that there was decrease in *E. Faecalis* CFU from pre-treatment to post irrigation in tested groups. Intra group comparison was statistically significant (P<0.001). Inter group comparison was non-significant for pre-treatment but statistically considerable for post irrigation comparison. There was lesser CFU in sodium hypochlorite group followed by triphala, neem, turmeric and maximum with green tea group.

An essential component of endodontic treatment is the application of irrigating solutions. Through a flushing effect, the irrigants help remove dentin chips, necrotic tissue and germs from the root canal. Irrigants assist keep infected soft and hard tissue from becoming packed apically in the root canal [1]. As endodontic irrigants, a number of natural extracts with demonstrated antibacterial effectiveness against *E. Faecalis*, such as Arctiumlappa, triphala, green tea polyphenols, liquorice, *etc.*, have been examined [13]. The bacteria chosen for this study were *E. Faecalis* because they are clinically linked to endodontic infection. The results of this study show that NaOCl is the best antibacterial, followed by triphala, neem and turmeric. NaOCl is the most common irrigants used in endodontics and it is thought to be the best because it kills bacteria, breaks down proteins and oxidises and hydrolyses substances [4]. Because of this, it was used as the study's control group. Mathew *et al.* tested how well a plant extract made in the study country called "EndoPam" worked *in vitro* compared to common endodontic irrigants at cleaning root canals. They came to the conclusion that the trial product worked just as well as regular irrigants at lowering the number of microbes [2]. Esmail *et al.* studied how well two natural plants extracts (Neem and tea tree oil, or TTO) kill microbes and two chemical root canal irrigations (sodium hypochlorite (NaOCL) and chlorhexidine) do at cleaning out the root canals. According to their findings, the studied herbal extract shows promise as an irrigants [1]. Researchers led by Afshan looked at how well neem leaf extract, *Morinda citrifolia* and water killed the bacteria *E. Faecalis*. They came to the conclusion that neem leaf extract was the most effective at killing *E. Faecalis*, while saline was the least effective [9]. From their study, Divya *et al.* came to the conclusion that triphala is a better antimicrobial than sterile water when used as a root canal irrigants [14].

Shalan compared how well two herbal extracts, 2.5% sodium hypochlorite and saline got rid of *E. Faecalis* germs. They came to the conclusion that all of the herbal irrigation treatments worked to get rid of *E. Faecalis* and could be used instead of NaOCL [15]. Ganesh *et al.* studied how root canal irrigants made from herbs and non-herbal ingredients killed *E. Faecalis*. They found that the nonherbal group (QMixTM 2 in 1, Endoseptone, Bio pure mixture of tetracycline, acid and detergent (MTAD)) had the strongest antimicrobial effects. On the other hand, the herbal group (*Morinda citrifolia* juice, Triphala juice and Coconut milk) also had significant drops in CFU counts. The group that didn't use herbs had the strongest antibacterial effects against *E. Faecalis* [4]. Researchers led by Kumar and others tested how well garlic, lemon and guava leaf worked against *E. Faecalis* as antimicrobial root canal irrigants. In the end, they found that herbal products worked better than 5% sodium hypochlorite against *E. Faecalis* [16]. Babaji *et al.* used sodium hypochlorite (NaOCl) to evaluate the antibacterial activity of herbal root canal irrigants (Azadirachta indica extract, Aloe vera and *Morinda citrifolia*) and discovered an inhibitory zone against *E. Faecalis* with the herbal irrigants they tested [17]. According to Gupta-Wadhwa *et al.* *Ocimum sanctum*, *Syzygium aromaticum* and *Cinnamomum zeylanicum* were effective against *Enterococcus faecalis* [18]. As a root canal irrigants, Nagaveni *et al.* discovered that various doses of chlorhexidine (CHX) and Aloe Vera extracts were effective against *E. Faecalis* [19]. Shalan assessed efficacy of turmeric irrigants and 25% propolis irrigants *E. Faecalis* and concluded that, they had antibacterial efficacy against *E. Faecalis* and can be used as irrigants [15]. Daga *et al.* concluded that, Sodium hypochlorite proved to be a better root canal irrigants followed by propolis, neem and miswak and were effective against *E. Faecalis* [20]. All the above studies had proved that, various herbal root canal irrigants are effective against *E. Faecalis* and can be used as alternative root canal irrigants. We found promising result from the tested herbal root canal irrigants which can be used alternative to sodium hypochlorite. Herbal root canal irrigants are cost effective easily available with antimicrobial properties with lesser side effects [9].

## Conclusion:

The tested herbal irrigants had antibacterial efficacy against *E faecalis*. Hence, these herbal irrigants are alternative to sodium hypochlorite.

## Figures and Tables

**Table 1:** *Enterococcus faecalis* CFU to different endodontic irrigants

**Group**	**Pre-treatment (CFU/ml 105)**	**post irrigation (CFU/ml 105)**	**p**
Group I- sodium hypochlorite (Control)	168.36±18.56	7.35±3.54	0.001
Group II- triphala	169	15	0.001
Group III- neem	165	35	0.001
Group V- turmeric	164	48	0.001
Group V- green tea	167	55	0.001
P	0.74	0.001	
